# Supplementary figures and images for: Form, function, and divergence of a generic fin shape in small cetaceans
Source: PLoS One. 2021 Aug 11;16(8):e0255464. doi: 10.1371/journal.pone.0255464 (PMC8357180; doi:10.1371/journal.pone.0255464)

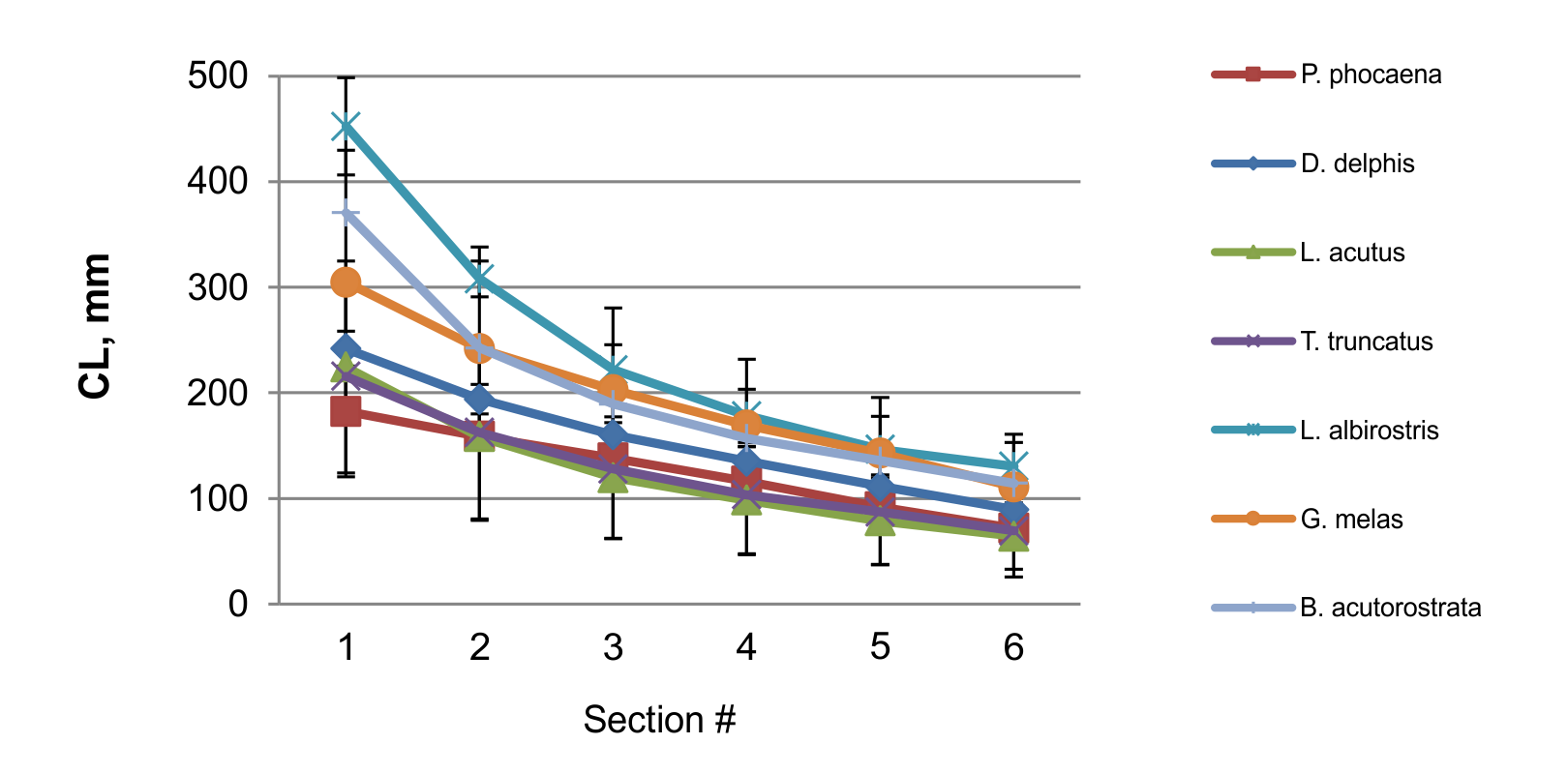

Supplement: S1 Fig — (TIF) [file pone.0255464.s001.tif]

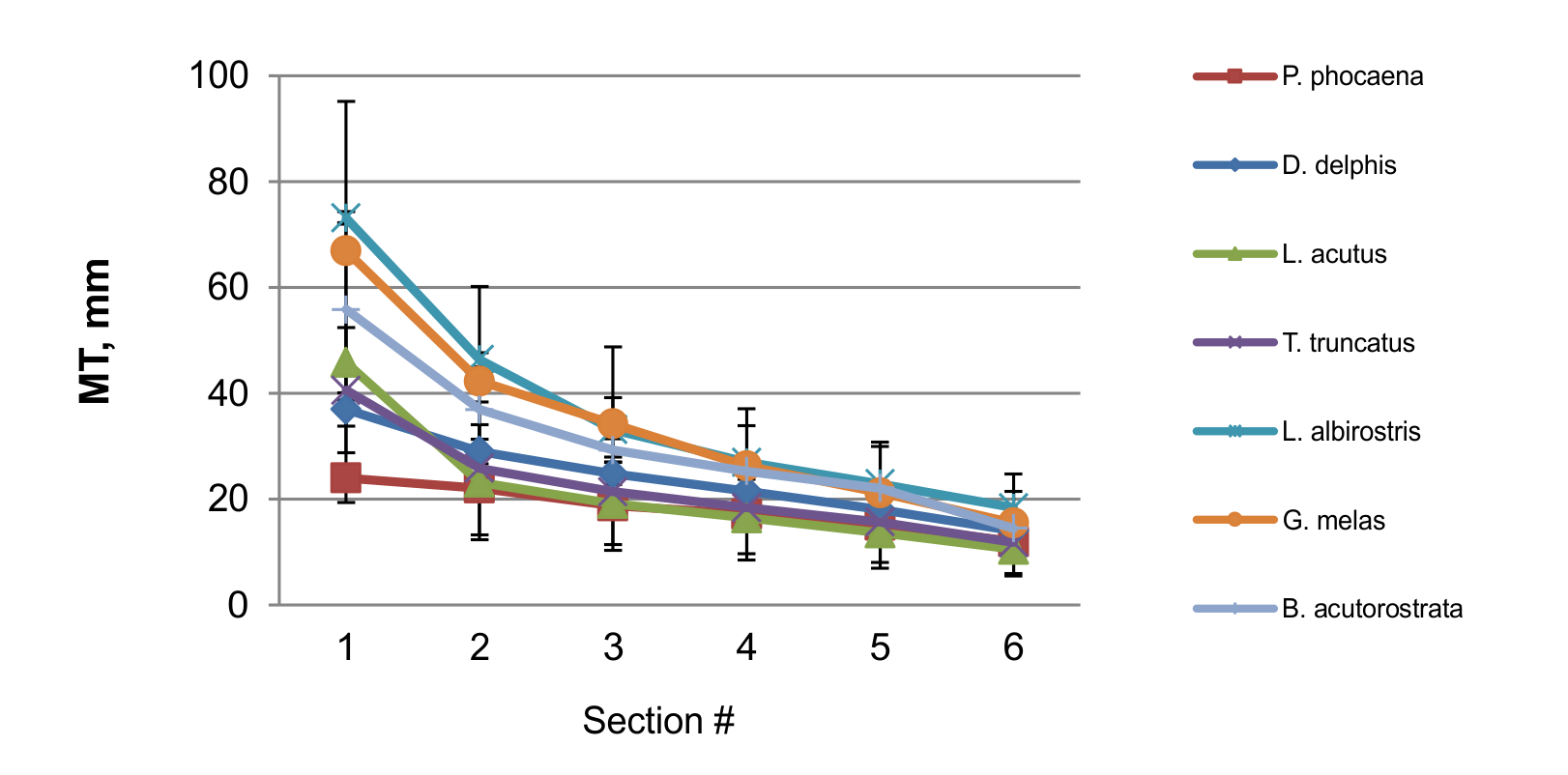

Supplement: S2 Fig — (TIF) [file pone.0255464.s002.tif]

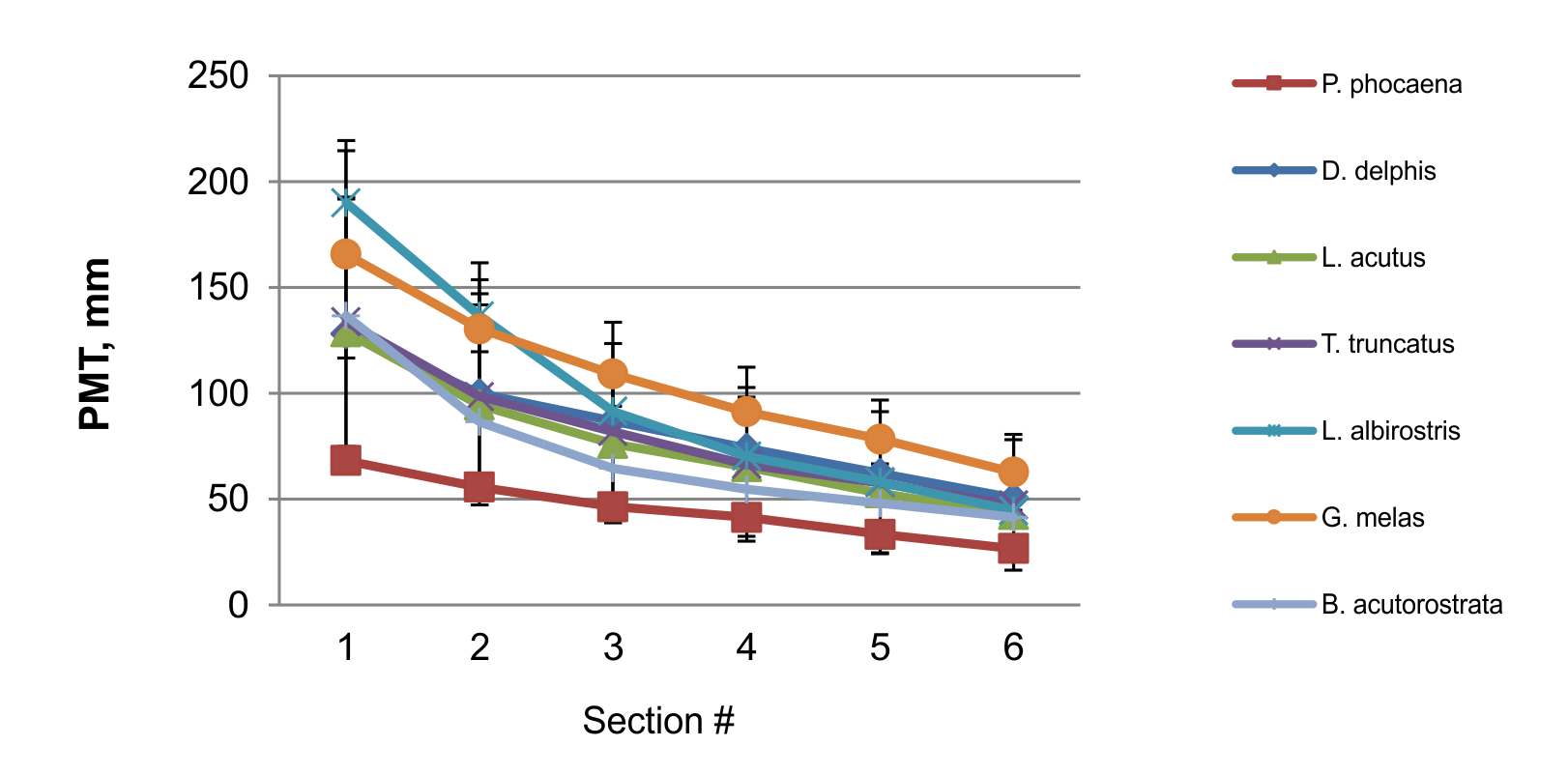

Supplement: S3 Fig — (TIF) [file pone.0255464.s003.tif]

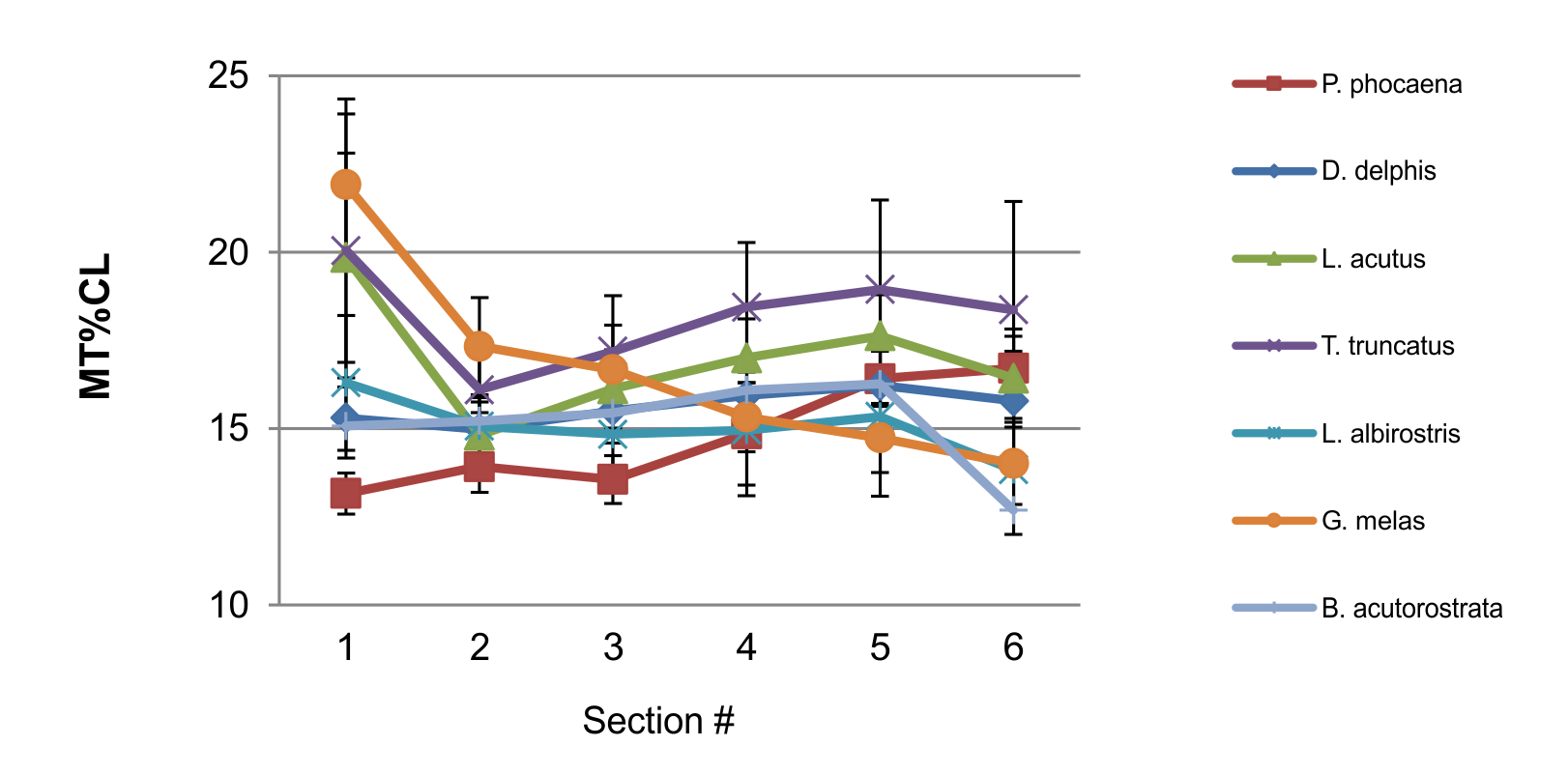

Supplement: S4 Fig — (TIF) [file pone.0255464.s004.tif]

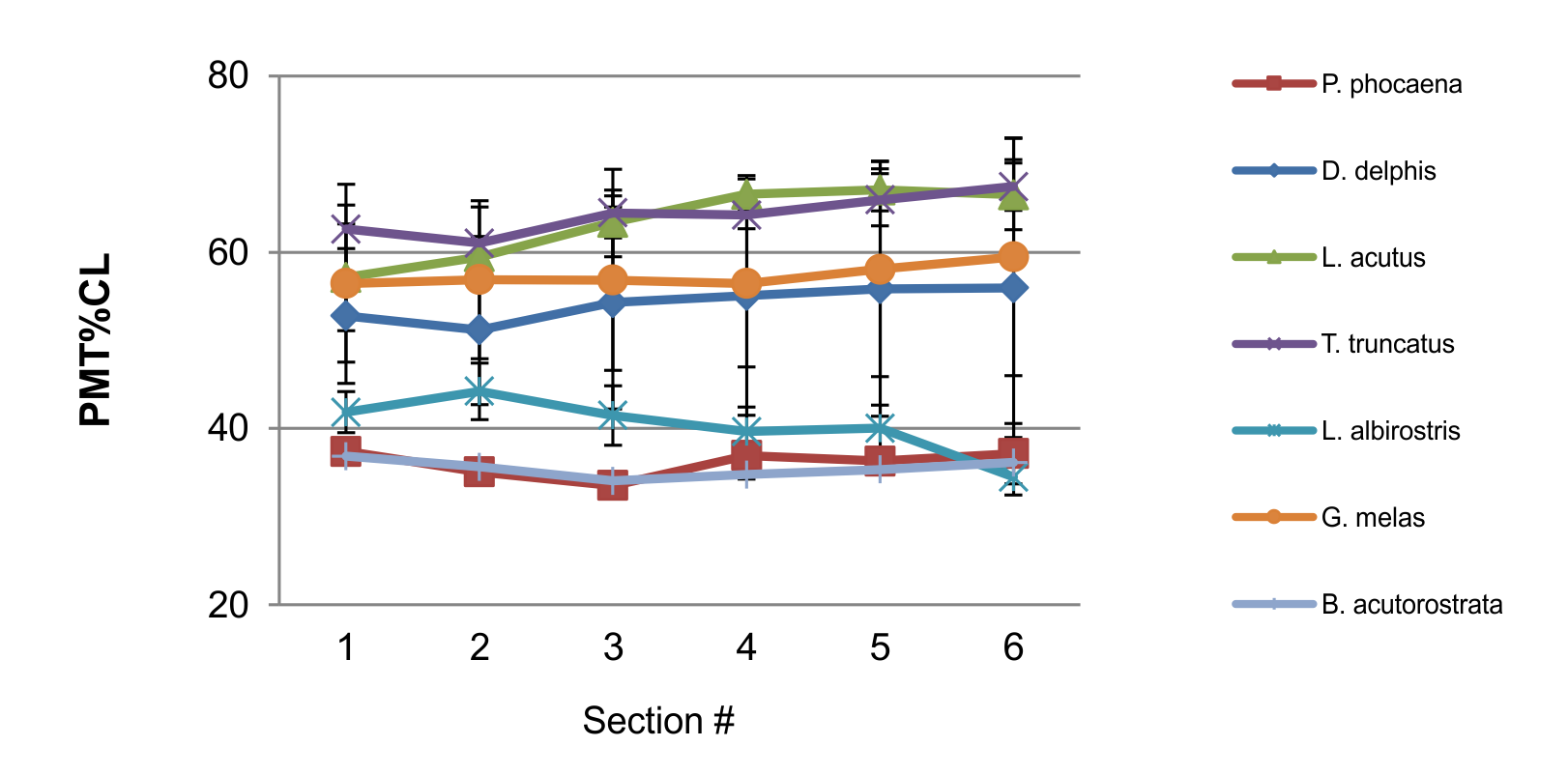

Supplement: S5 Fig — (TIF) [file pone.0255464.s005.tif]

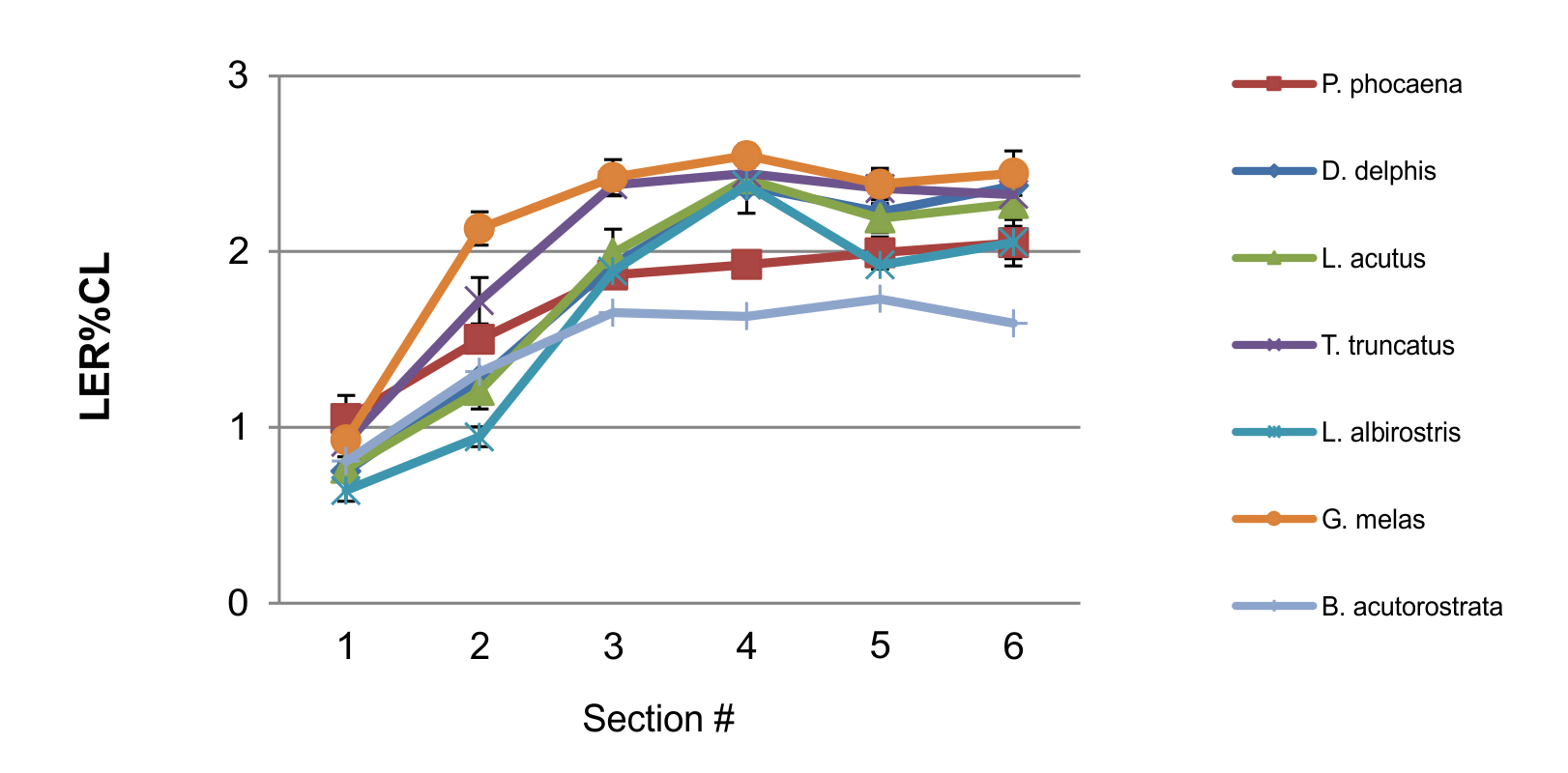

Supplement: S6 Fig — (TIF) [file pone.0255464.s006.tif]

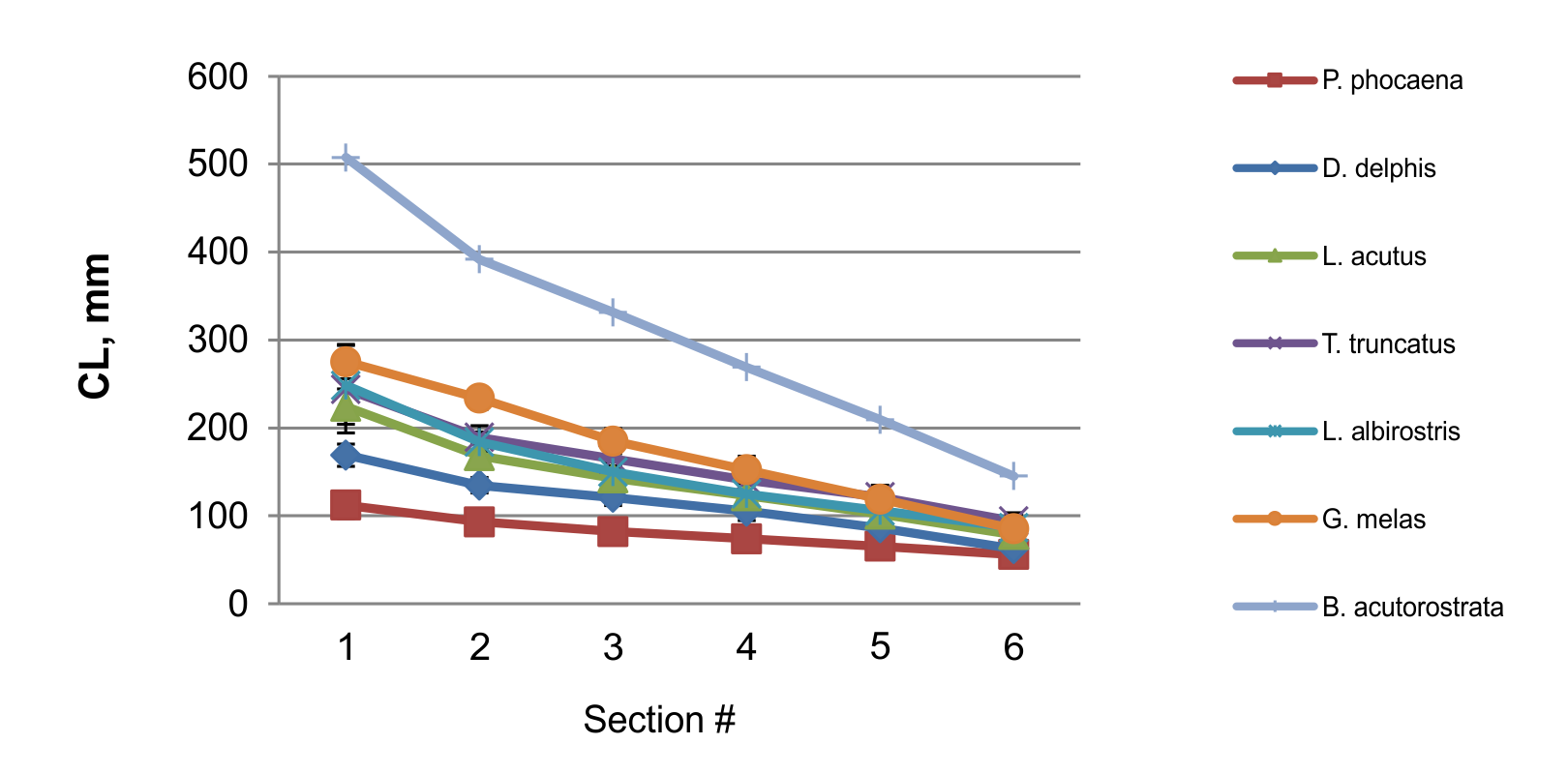

Supplement: S7 Fig — (TIF) [file pone.0255464.s007.tif]

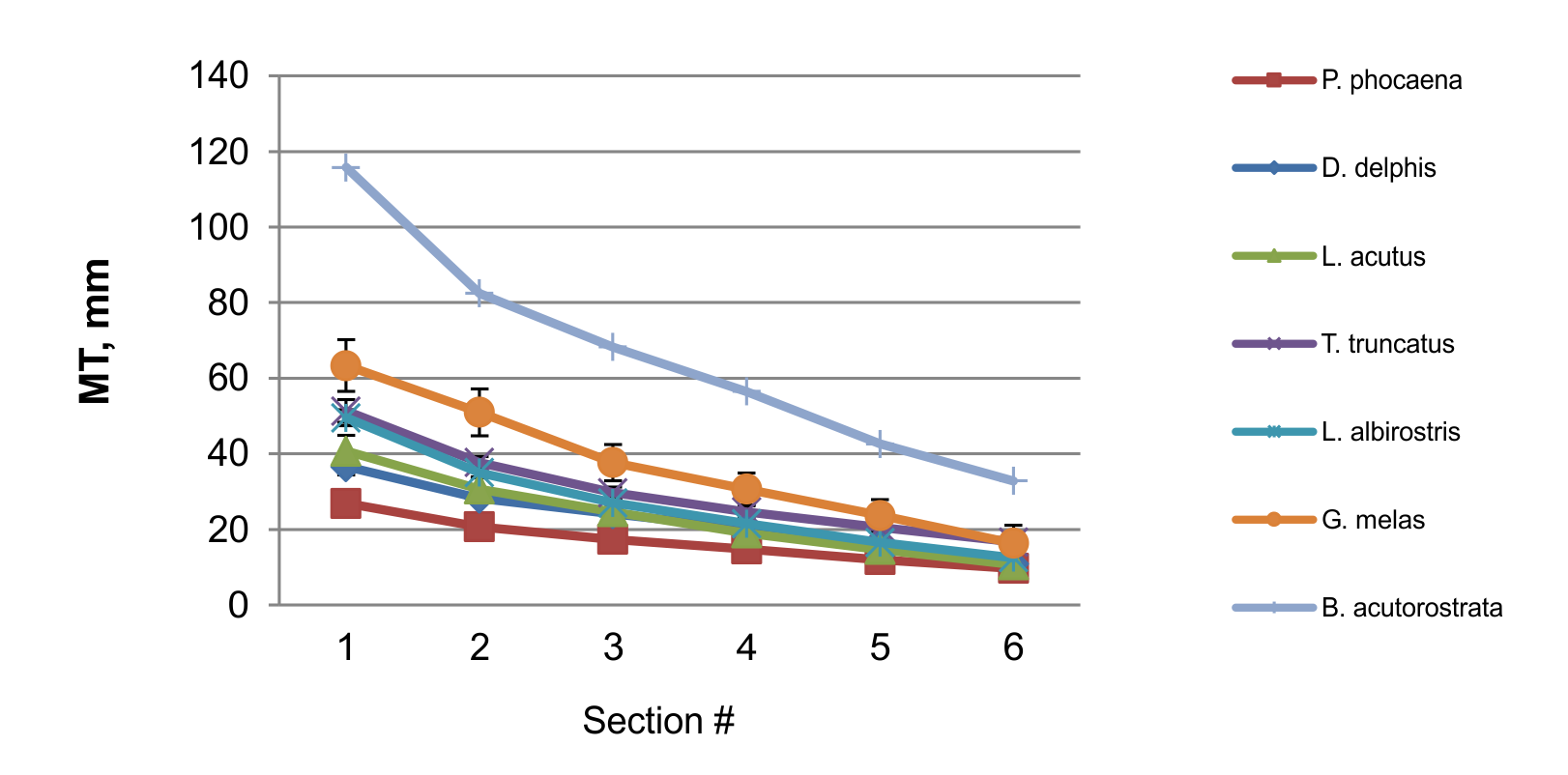

Supplement: S8 Fig — (TIF) [file pone.0255464.s008.tif]

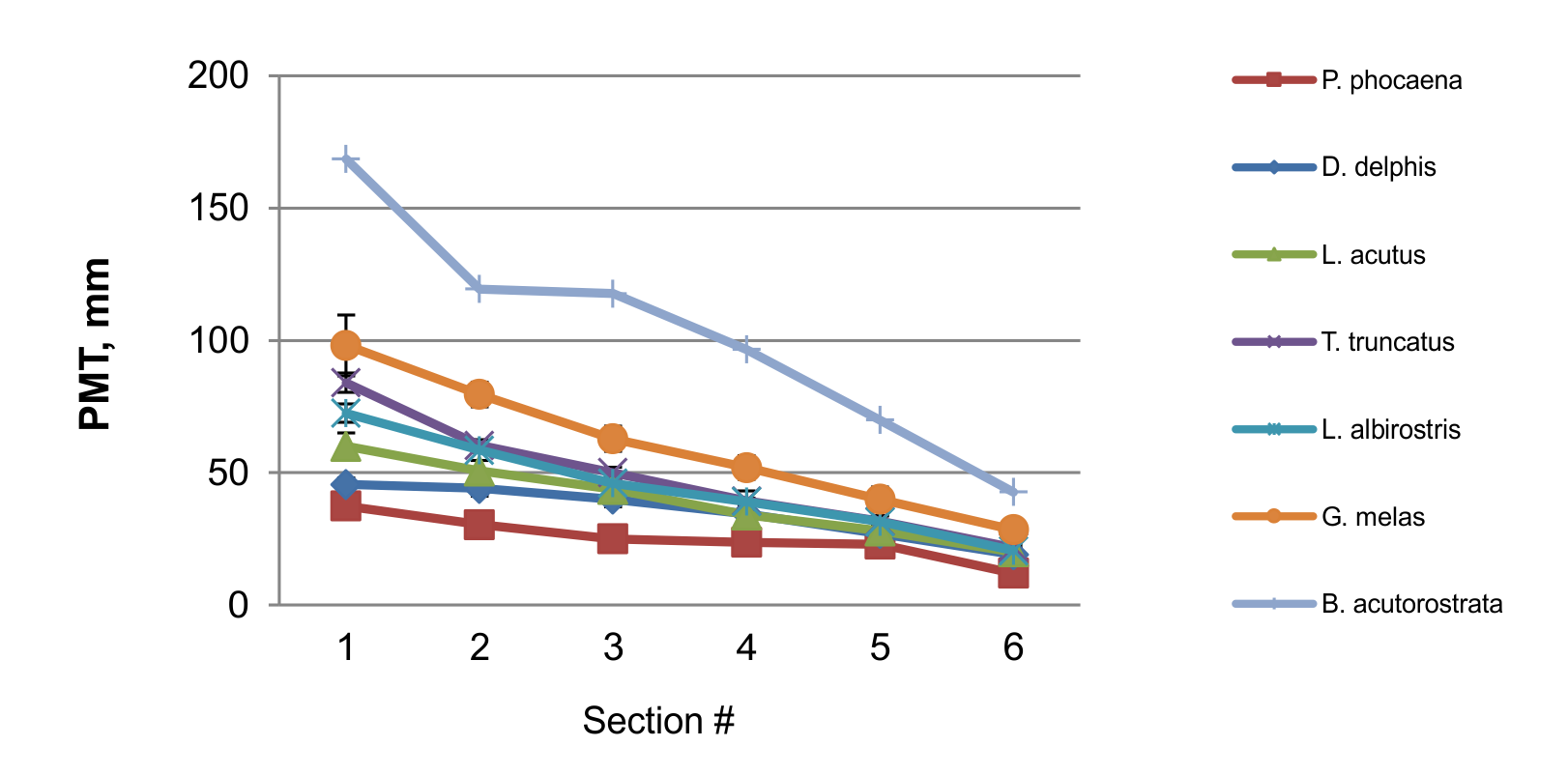

Supplement: S9 Fig — (TIF) [file pone.0255464.s009.tif]

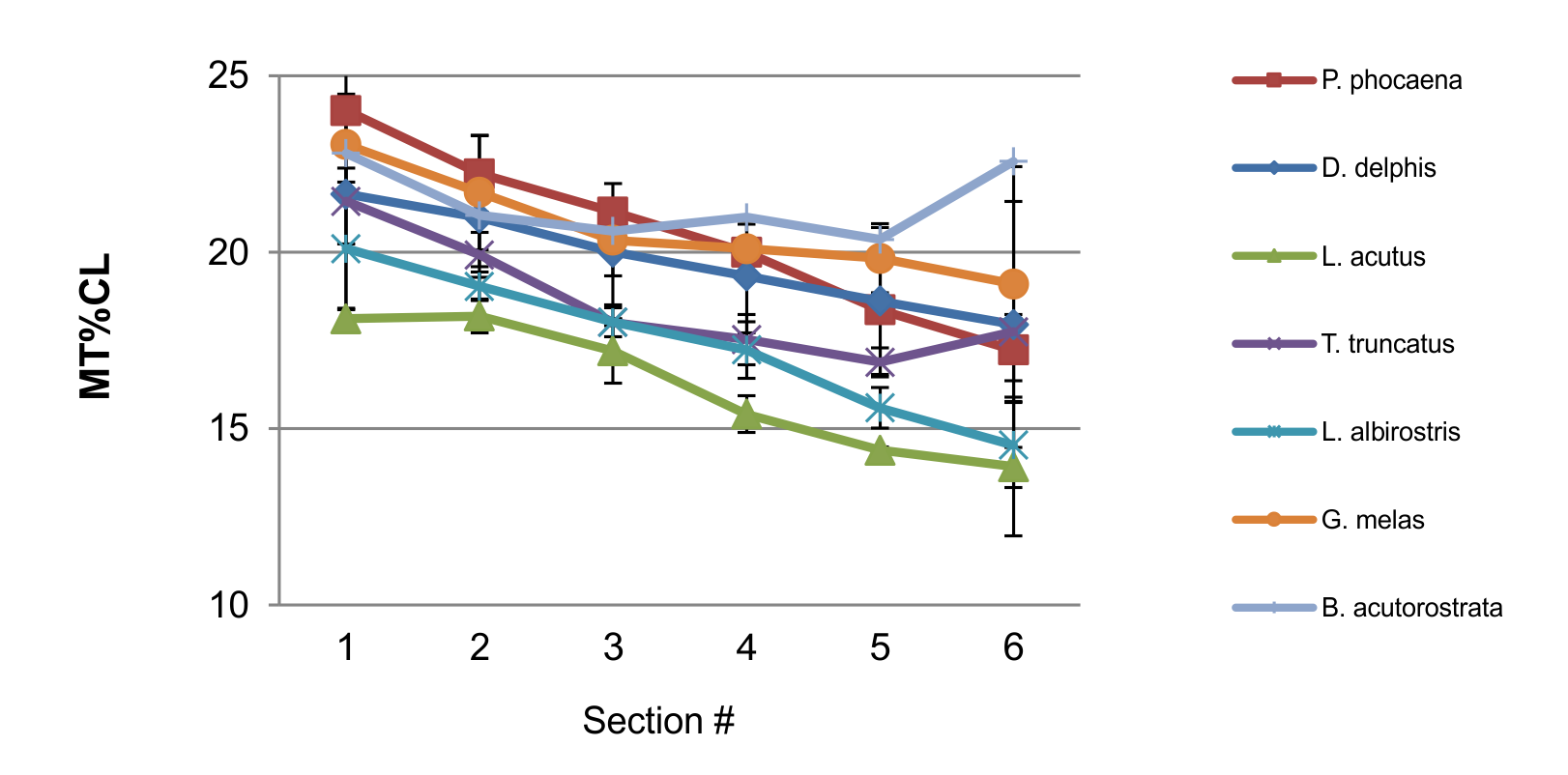

Supplement: S10 Fig — (TIF) [file pone.0255464.s010.tif]

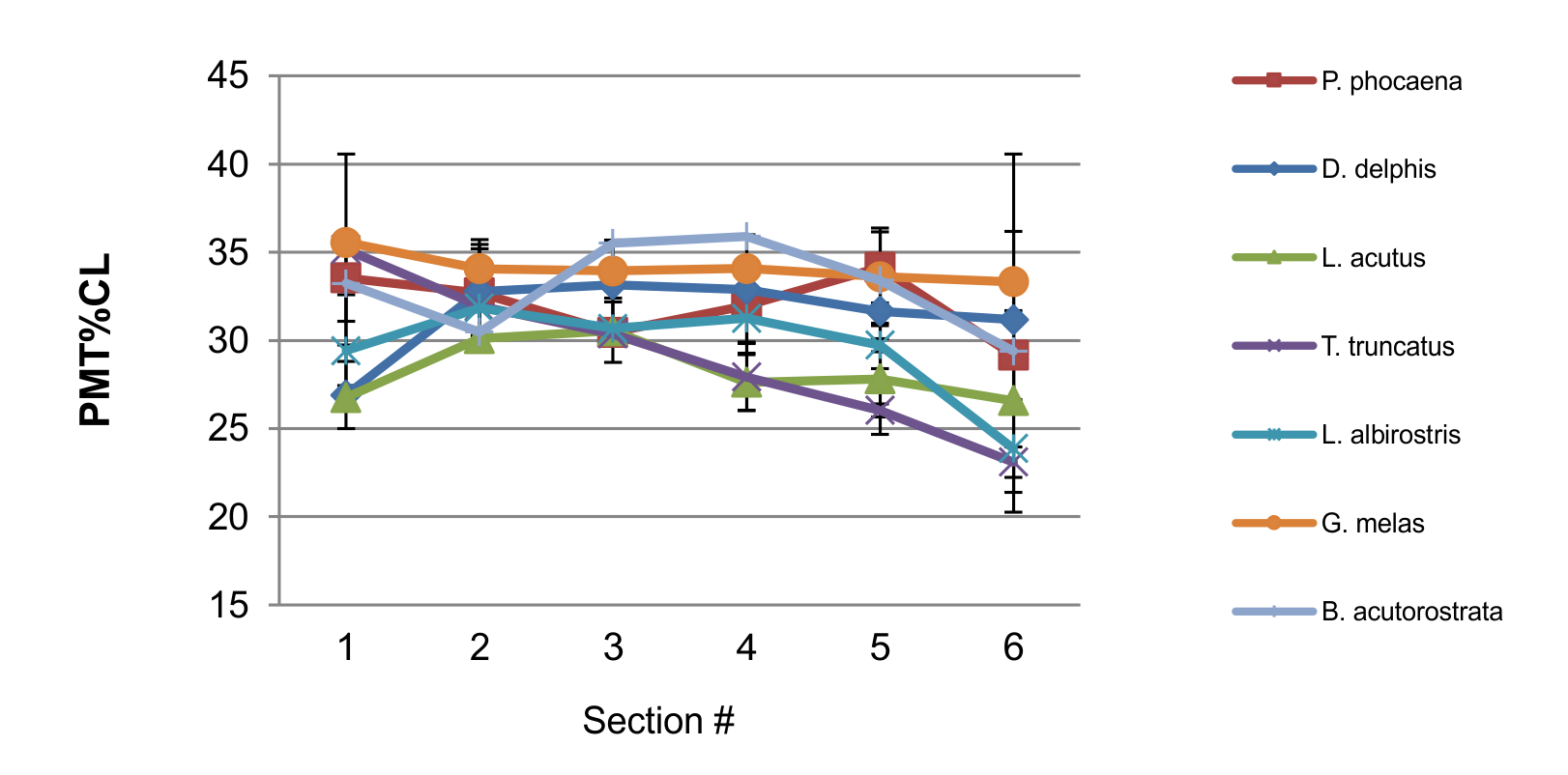

Supplement: S11 Fig — (TIF) [file pone.0255464.s011.tif]

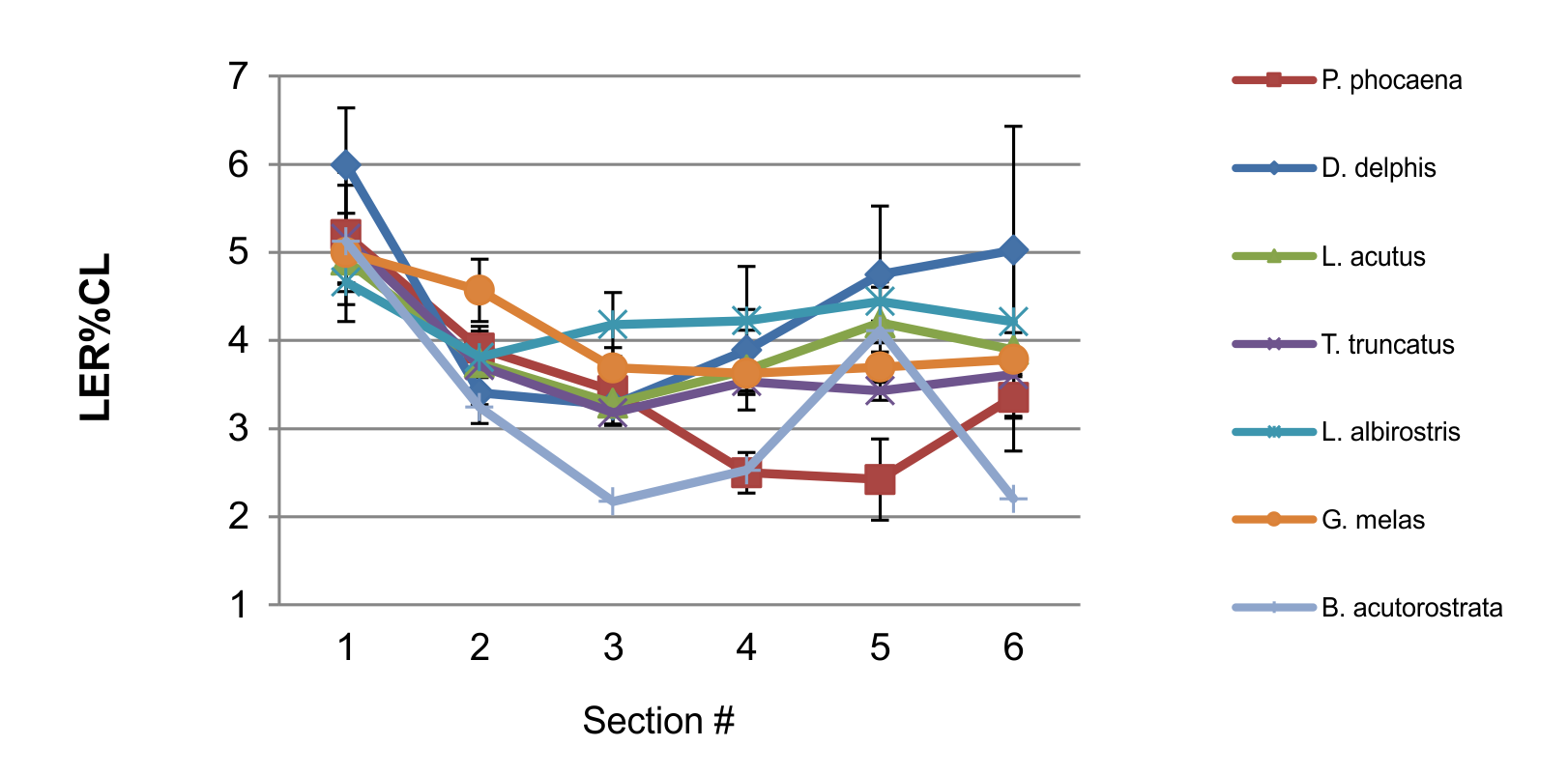

Supplement: S12 Fig — (TIF) [file pone.0255464.s012.tif]

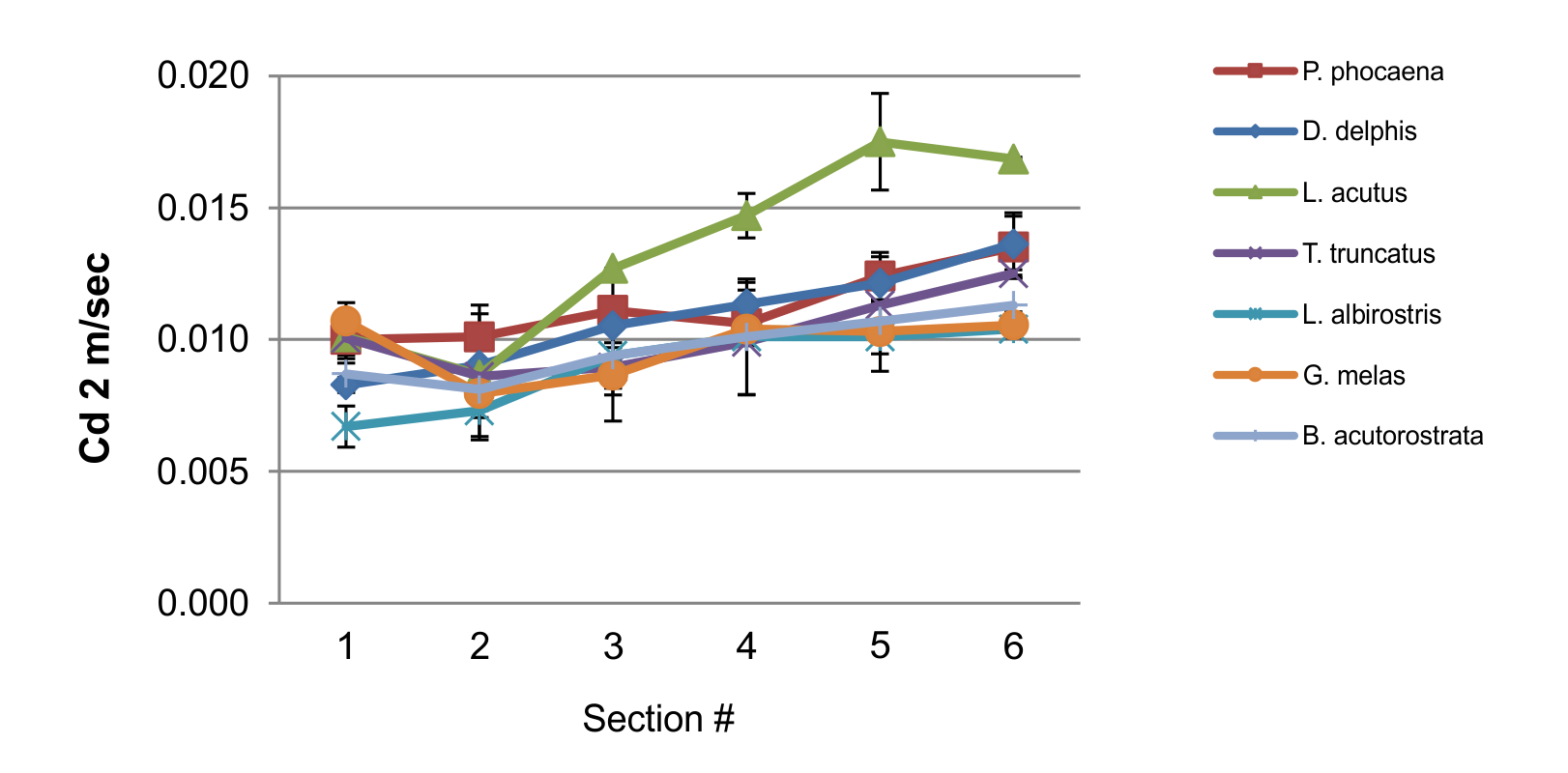

Supplement: S13 Fig — (TIF) [file pone.0255464.s013.tif]

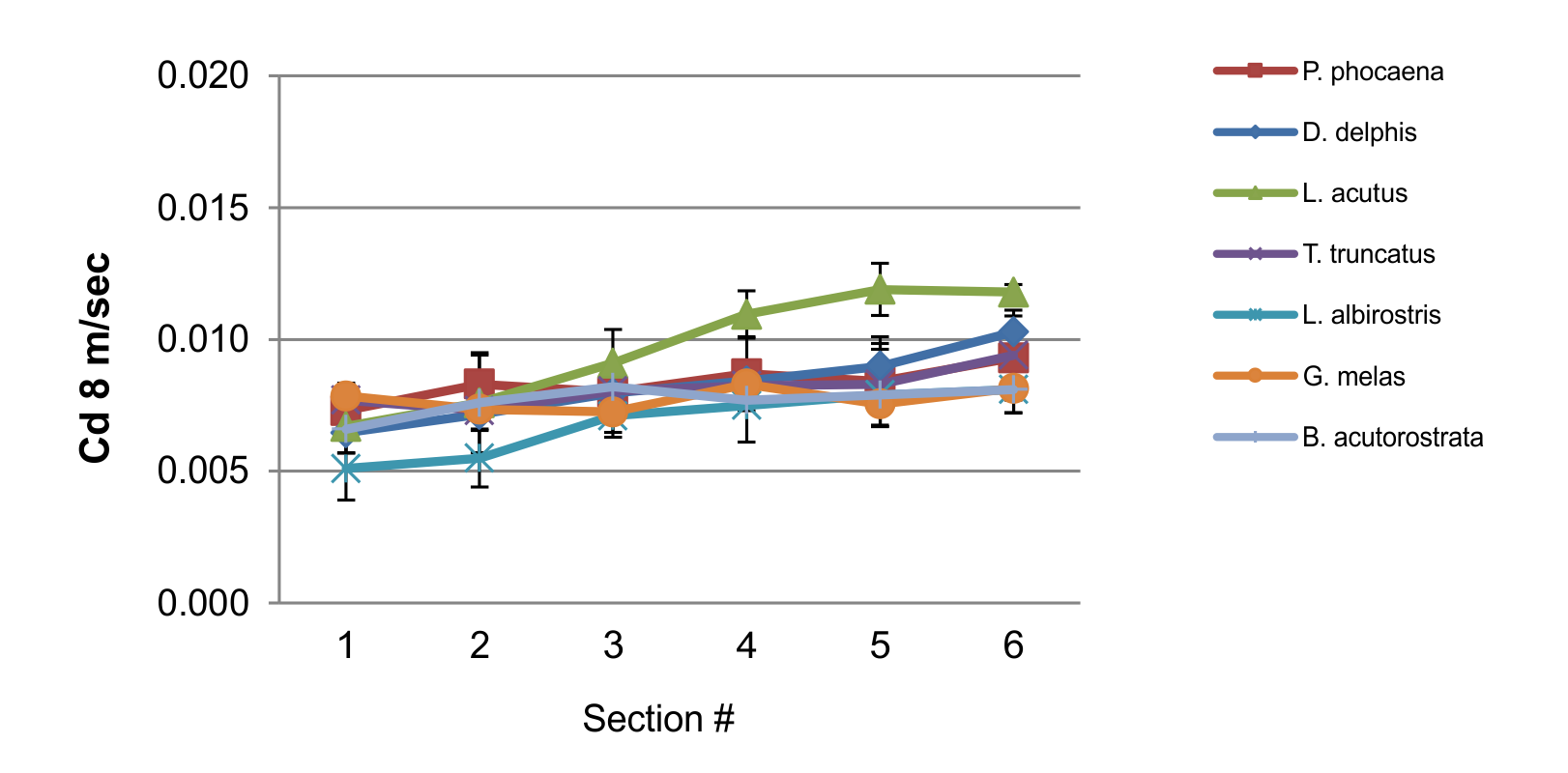

Supplement: S14 Fig — (TIF) [file pone.0255464.s014.tif]

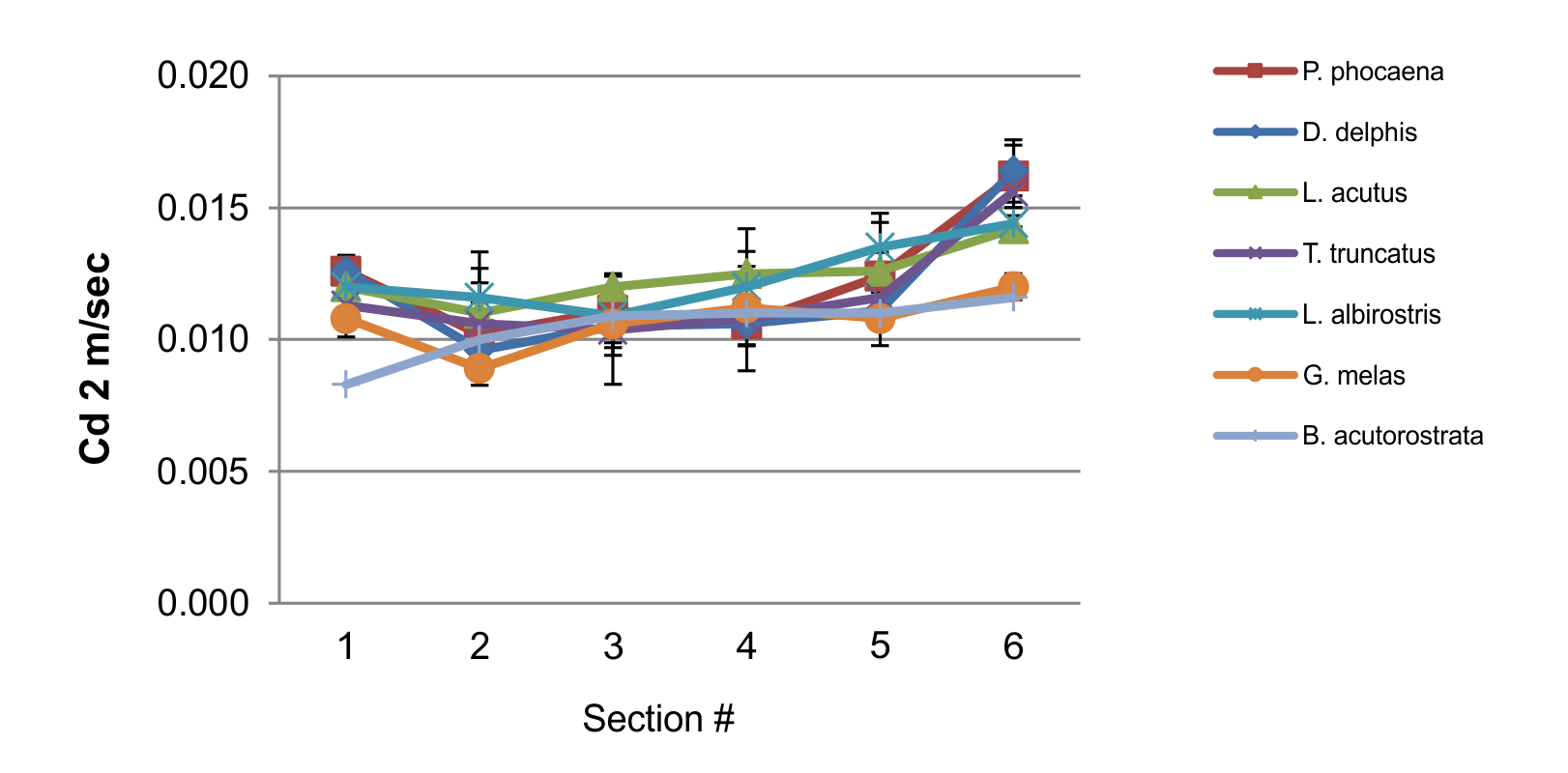

Supplement: S15 Fig — (TIF) [file pone.0255464.s015.tif]

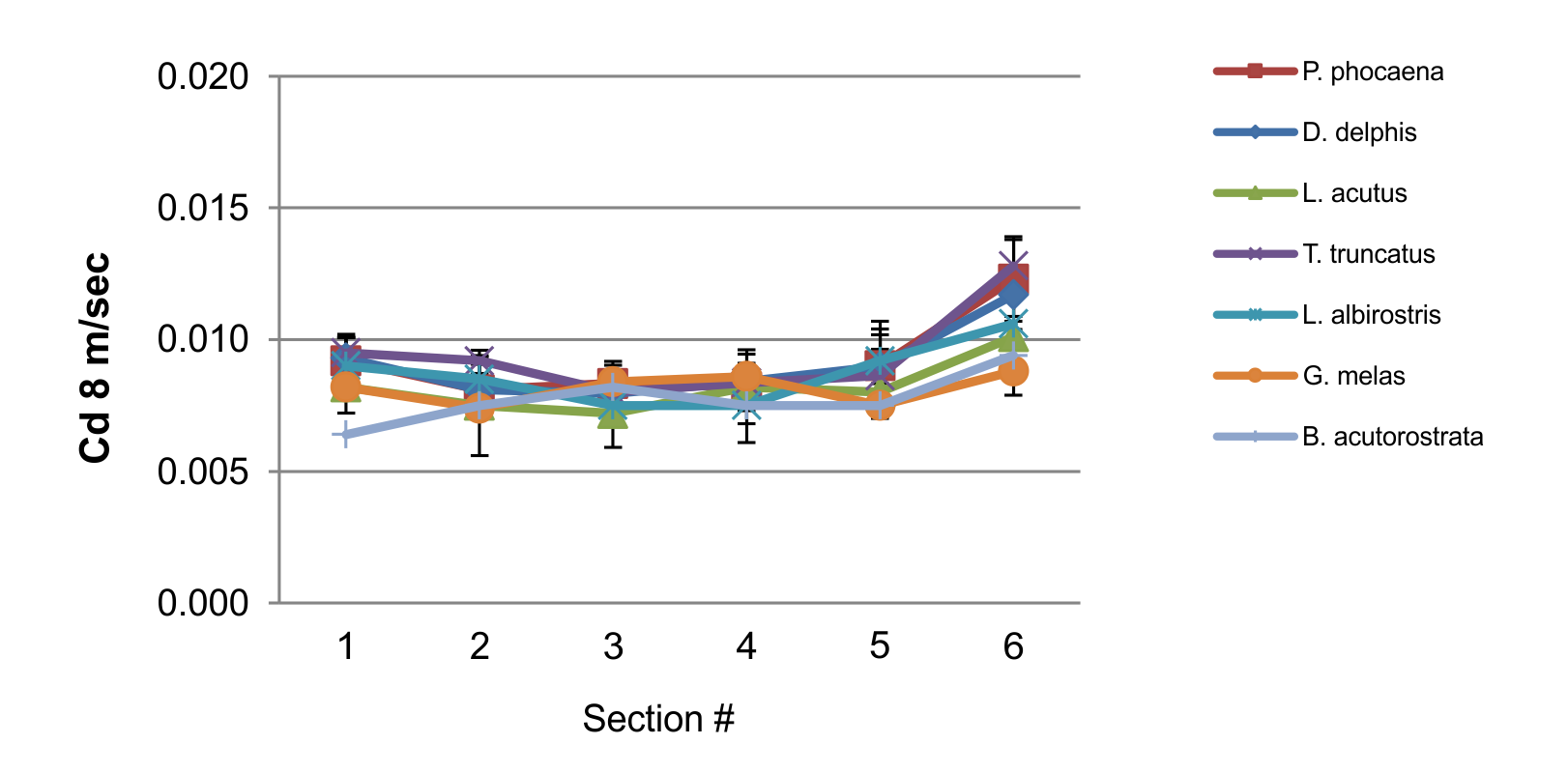

Supplement: S16 Fig — (TIF) [file pone.0255464.s016.tif]
